# Is air pollution negatively associated with physical fitness?—A cross-sectional study in 174,246 Chinese students

**DOI:** 10.1371/journal.pone.0336417

**Published:** 2025-11-06

**Authors:** Weixin Chen, Jiaming Yan, Zhenxing Kong, Yuliang Sun, Wenfei Zhu, Jie Kang

**Affiliations:** 1 School of Physical Education, Shaanxi Normal University, Xi’an, China; 2 Key Laboratory of Exercise and Physical Fitness (Beijing Sport University), Ministry of Education, Beijing, China; National Autonomous University of Mexico Institute of Geophysics: Universidad Nacional Autonoma de Mexico Instituto de Geofisica, MEXICO

## Abstract

**Objectives:**

Air quality in China has become an increasing concern, its association with physical fitness remains unclear. This study represents one of the largest nationwide investigations of this association, leveraging data from 174,246 students aged 7–22 years across 30 provinces.

**Methods:**

Annual concentrations of PM₂.₅, PM₁₀, SO₂, NO₂, CO, O₃, and the Air Quality Index (AQI) were obtained from the Tracking of Atmospheric Pollution in China dataset. Physical fitness was evaluated through a comprehensive set of field-based tests covering anthropometric, cardiopulmonary, flexibility, muscular strength, and endurance. Associations were examined using generalized linear models with progressive adjustments: Model 1 controlled for demographic factors (age, sex, residence, province), Model 2 additionally accounted for physical activity and parental factors, and Model 3 further incorporated temperature and humidity.

**Results:**

After adjusting for covariates, each 1 μg/m³ increase in PM₂.₅ and PM₁₀ was associated with decreases in physical fitness scores of 0.18 [95% CI: −0.22, −0.14] and 0.12 [−0.16, −0.08] points, respectively. SO₂, O₃, and CO showed similar negative associations, with reductions of 0.42 [−0.47, −0.38], 0.21 [−0.25, −0.16], and 0.16 [−0.20, −0.11] points, respectively. In contrast, NO₂ exhibited a positive association, with an increase of 0.29 [0.25, 0.33] points per 1 μg/m³. AQI was also inversely related to fitness, decreasing scores by 0.17 [−0.21, −0.13] points per 1-unit increase.

**Conclusions:**

Ambient air pollution is adversely associated with physical fitness among Chinese children, adolescents, and young adults, highlighting the importance of air quality improvement strategies for youth health. Future longitudinal studies are warranted to strengthen causal inference.

## 1. Introduction

With the rapid pace of industrialization and urbanization, the impact of air pollution has become increasingly prominent. Based on air quality monitoring and scientific evidence of the impact of air pollution on human health, World Health Organization (WHO) has published air quality guidelines for urban cities worldwide [1]. However, according to the WHO, nearly 99% of the global population breathes air that exceeds the recommended pollution limits [2], highlighting the widespread severity of the issue. In China, a growing number of studies have applied machine learning and statistical models to monitor and predict air pollution, including PM₂.₅ and O₃ concentrations, in cities such as Liaocheng [3,4], Shanghai [5], Jinan [6], Hohhot [7], and Dezhou [8]. While these advancements have contributed to more targeted and timely pollution control strategies, air quality in many regions remains suboptimal. In some areas, pollution levels have even worsened in recent years, posing ongoing risks to public health [9]. Consequently, reducing air pollution has become a national priority in the world’s largest developing economies, including China [10].

Physical fitness is widely regarded as a comprehensive indicator of bodily functions, encompassing systems such as the skeletomuscular, cardiorespiratory, circulatory, neurological, and endocrine systems. High levels of physical fitness, reflected in cardiopulmonary endurance, muscle strength, and flexibility, signify the optimal functioning of these systems and serve as a reliable marker of overall health [11]. Physical fitness tests effectively assess these indicators, and using such tests to evaluate the impact of air pollution on general health is both scientifically valid and practically feasible.

However, emerging evidence indicates that exposure to air pollutants impairs multiple dimensions of physical fitness. Ozone (O_3_) and particulate matter with an aerodynamic diameter of ≤10 μm (PM_10_) are linked to poorer lung function, elevated blood pressure and reduced cardiorespiratory performance, which translate into slower times in endurance (800 m/1000 m run), [12–14] and sprint events (8 × 50 m shuttle run and 50 m sprint) [15]. Particulate matter with an aerodynamic diameter of ≤2.5 μm (PM_2.5_) and nitrogen dioxide (NO_2_) exposure increases the risk of sarcopenia [16] and weakens grip strength, undermining muscle-strength tests like pull-ups and sit-ups [17,18], PM₂․₅, PM₁₀ and sulfur dioxide (SO_2_) also promote oxidative stress and adipose inflammation [19], contributing to higher body-mass index and impact the physical fitness of Chinese students. While existing research confirms air pollution negatively impacts physical health markers relevant to fitness, there is still a lack of systematic research on the relationship between air pollution and physical fitness, especially in terms of its effects on Chinese children, adolescents, and young people at different pollution levels. This study contributes to the existing literature by examining how different levels of air pollution affect multiple dimensions of physical fitness—such as endurance, muscular strength, and body composition—specifically among Chinese children, adolescents, and young adults.

The present study addresses this gap by analyzing 174,246 students aged 7–22 from 30 Chinese provinces, combining standardized physical fitness tests with concentrations of PM₂.₅, PM₁₀, SO₂, NO₂, CO and O₃. We hypothesized that air pollution is negatively associated with physical fitness in this population. This study fills a critical gap in existing research and has the potential to enhance public awareness of air pollution’s harmful effects on health, while promoting environmental protection and health guidelines.

## 2. Methods

This cross-sectional study was conducted between 01/09/2019 and 01/02/2020 in major cities across China, examining the association between ambient air quality and physical fitness. Air pollutant concentrations, including AQI, PM₂.₅, PM₁₀, SO₂, NO₂, O₃, and CO, were obtained from multi-source datasets integrating ground-based observations, satellite remote sensing, emission inventories, and model simulations (http://tapdata.org.cn). Meteorological data (monthly average temperature and humidity) were sourced from the China Meteorological Data Service Centre.

### 2.1. Study population

**Fig 1:**
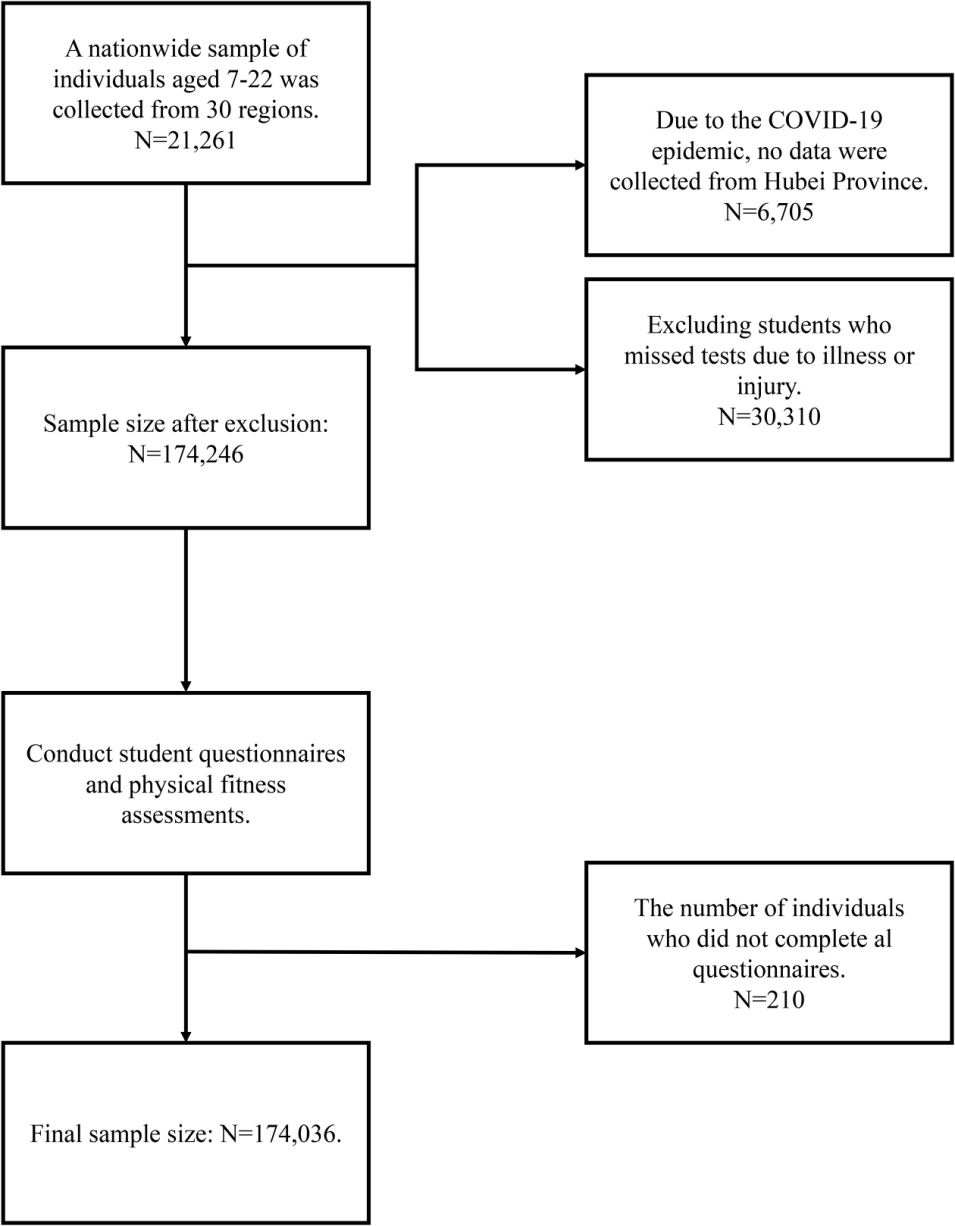
Selection and screening of study participants.

Participants came from the 2019 National Student Physical Fitness and Health Survey and the National Student Physical Fitness Test conducted by the Ministry of Education of the People’s Republic of China. Participants were recruited from 31 provinces, municipalities, and autonomous regions in mainland China (excluding Hong Kong, Macau, and Taiwan). Due to the COVID-19 pandemic, data from Hubei Province were not collected, reducing the final number of provinces in the study to 30. In total, 174,246 students completed both the questionnaire and physical fitness test; after excluding incomplete records, 174,036 students aged 7–22 years were included, ranging from third-year primary school to fourth-year university (Fig 1). The study included physical exams, standard disease screenings, and a lifestyle questionnaire. Based on GDP rankings, cities within each province were classified into four economic tiers [20]. Sampling followed GDP-based economic tier classification: within each province, cities were classified into four economic tiers, and one city per tier was randomly selected. For centrally governed municipalities, one urban and one suburban district were selected; for other cities, two subordinate administrative units (counties, districts, cities, or banners) were randomly chosen. This study was approved by the Ethics Committee of Shaanxi Normal University (202316016) and complied with the standards of the Declaration of Helsinki. All participants (or their legal guardians, if the participant was a minor) provided written informed consent at the time of data collection for their data to be used in research.

### 2.2. Air pollution

We used daily average concentrations of AQI (unit), PM _2.5_ (µg/m³), PM_10_ (µg/m³), SO_2_ (µg/m³), NO_2_ (µg/m³), O_3_ (µg/m³), and CO (ppm) from 01/09/2019–01/02/2020 were used based on the timing of the cross-sectional survey period, which physical fitness tests were carried out during this period, in accordance with the Chinese National Survey on Students’ Constitution and Health (CNSSCH) schedule. Due to privacy constraints preventing home address collection, exposure was assigned based on school location, as students spend most waking hours on campus. This approach may cause exposure misclassification, as personal exposures could differ from school-level concentrations, but it remains the most feasible option in large-scale surveys. Each school’s addresses were geocoded, and corresponding pollutant concentrations were assigned. Monthly average temperature and humidity data were obtained from the China Meteorological Data Service Centre for 124 cities across 30 provinces [21]. Temporal alignment of exposure and testing. We summarized daily pollutant concentrations to the testing-month mean for each school; when the exact testing month was unavailable, we used the survey-period mean for that school (Sep 2019–Feb 2020). Because testing months were not uniformly available, exposure and outcome windows may not fully coincide at the monthly or seasonal scale; this non-differential misalignment would be expected to attenuate associations toward the null.

### 2.3. Physical fitness

Physical fitness was assessed using the Chinese National Student Physical Fitness Test (2014 edition) [22], which includes indicators of body composition (BMI) [23], cardiopulmonary function (FVC), flexibility (sit-and-reach), muscular strength (sit-ups or pull-ups, standing long jump), speed (50m sprint), agility (50m × 8 round-trip run), endurance (800m for girls, 1000m for boys), and coordination (1-min rope skipping). Full Physical fitness test protocols are provided in S1 Text. All testers received standardized training, instruments were sourced from the same certified supplier, and uniform quality-control procedures were followed. Data were double-entered, cross-checked, and verified before scoring. Finally, the measurements were assigned corresponding scores. The testing items and scoring criteria differ across grade levels, as shown in Table 1.

**Table 1:** The content of the test and the weighting of the scores for each academic level.

Test Subject	Individual indicators	Weights %
Primary 1 to College	BMI	15
	FVC	15
Primary 1 and 2	50m sprint	20
	Sit-and-reach	30
	1-min rope skipping	20
Primary 3 and 4	50m sprint	20
	Sit-and-reach	20
	1-min rope skipping	20
	1-min sit-ups	10
Primary 5 and 6	50m sprint	20
	Sit-and-reach	10
	1-min rope skipping	10
	1-min sit-ups	20
	50m × 8 round-trip running	10
Middle School to College	50m sprint	20
	Sit-and-reach	10
	Standing long jump	10
	1-min sit-ups (Girls)/pull-ups (Boys)	10
	800 m running (Girls)/1000 m running (Boys)	20

### 2.4. Covariates

Demographic variables were obtained from the study questionnaire: age, sex(boys/girls), and residence (urban/rural). Lifestyle variables included exercise behaviour and parental attitudes toward physical activity were included. Average daily physical activity hours were calculated as: [(weekday hours × 5) + (weekend hours × 2)]/7.

According to the Chinese Physical Activity Guidelines (2021) [24], we categorised moderate-to-vigorous physical Activity into two categories: physical activity ≥1 hour per day and physical activity <1 hour per day. Similarly, we categorised outdoor activities into two categories: individuals who participated in outdoor activities for ≥2 hours per day and those who participated for <2 hours per day. Information on muscle-strengthening exercises was collected using the following question: “In the past seven days, how many days did you engage in muscle-strengthening exercises? (Including unarmed exercises such as squats, 1-min sit-ups, push-ups, pull-ups, etc., as well as exercises with dumbbells, elastic bands, and fitness equipment)” and categorised into two groups: ≥ 2 days/week, < 2 days/week. Parental support and preference were obtained via questionnaire. Regional covariates (annual average temperature and humidity) came from the China Meteorological Administration [25].

### 2.5. Statistical analysis

We applied generalised linear models (identity link, Gaussian distribution) to investigate the asscications between environmental pollutants and physical fitness score, standardising continuous variables to improve stability. This approach was chosen because the outcome variable was continuous and approximately normally distributed, and the identity link allowed direct interpretation of coefficients as score changes per unit pollutant concentration. The test items and scoring criteria varied according to grade level (Table 1). Three models with progressively more covariates based on the previous study [26]. Model 1: adjusted for age (numeric variable), sex (categorical variable), residence (categorical variable), and province (categorical variable); Model 2: further adjusted for moderate-to-vigorous physical activity and outdoor activity, muscle-strengthening exercise, parental support for physical activity, and parental preference for physical activity (all categorical variables); Model 3: further adjusted for temperature (numeric variable) and humidity (numeric variable). All pollutant variables had VIF < 2. Given the low proportion of missing values in the covariates, we excluded them to maintain dataset completeness and robustness for statistical analyses.

To examine whether associations differed by demographic and contextual factors, we conducted stratified analyses rather than fitting hierarchical or mixed-effects models. Although the dataset had a nested structure (students within schools, schools within cities/provinces), we did not adopt mixed-effects models due to substantial imbalance in sample sizes across clusters and because the primary aim was to estimate overall associations rather than to model between-cluster variance. Specifically, we stratified the dataset by sex (girls, boys), grade (primary, middle, high, college), and region (urban, rural), and fitted separate generalised linear models (GLMs) with an identity link and Gaussian distribution within each stratum. This approach enabled direct comparison of effect estimates across strata and facilitated interpretation of heterogeneity, including unexpected patterns such as the stronger positive association between NO₂ and physical fitness observed in girls. A comprehensive set of sensitivity analyses was performed to evaluate the robustness of our findings. Different time windows were used to calculate the AQI index and individual air pollution concentrations, using 3- to 5-year average concentrations (baseline survey) instead of the 1-year averages employed in the original analyses. All analyses were conducted in R (version 4.2.2) using the “glm” function from the “stats” package.

## 3. Results

The current study included 174,036 participants, as outlined in S1 Table. At baseline, the mean age was 15.13 ± 3.93 years, and 57.1% of the participants were boys. Furthermore, most participants (52.1%) resided in rural areas. The overall engagement in exercise was encouraging, except for muscle-strengthening exercises, where only 30.7% of participants engaged in them at least twice a week. Most parents (80.5%) supported physical activity for their children. Among these parents, 46.1% both liked exercise, 18.9% of fathers liked exercise, 9.9% of mothers liked exercise, and 25.1% of both parents did not like exercise.

**Fig 2:**
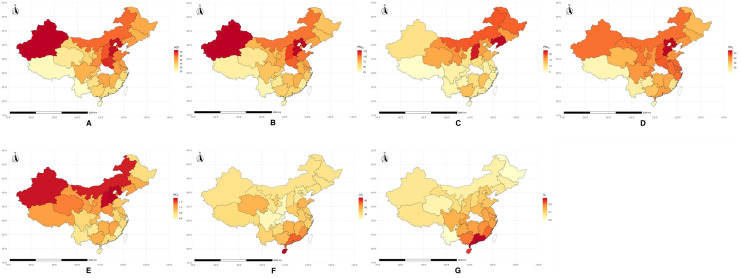
Distribution of AQI and Air pollutants indicators by provinces. A: Air Quality Index (AQI), B: CO (Carbon Monoxide), C: NO_2_ (Nitrogen Dioxide), D: O₃ (Ozone), E: PM_10_ (Particulate Matter 10 micrometers), F: PM_2.5_ (Particulate Matter 2.5 micrometers), G: SO_2_ (Sulfur Dioxide). Map rendered using public domain geographic data from Natural Earth (www.naturalearthdata.com). All map layers are CC0 licensed. Map generated with R (ggplot2 + sf).

S2 Table presents a detailed summary of AQI and air pollutants distribution across 30 provinces, and the distribution of AQI and air pollutants exposure for each province is represented in Fig 2. The data in this table reveals that Xinjiang recorded the highest AQI and PM_2.5_, while Hebei had the highest PM_10_ concentration. In contrast, Yunnan had the lowest AQI and PM_2.5_. For gaseous pollutants, Shanxi exhibited the highest SO_2_, and Shanghai the highest NO_2_. Fujian had the lowest CO, while Yunnan showed the highest O_3_ level. Regarding climate data, Hainan experienced the highest temperature, while Sichuan recorded the highest humidity, contrasting with Xizang’s lowest humidity. This highlights significant regional disparities in air quality and climate across the provinces.

**Fig 3:**
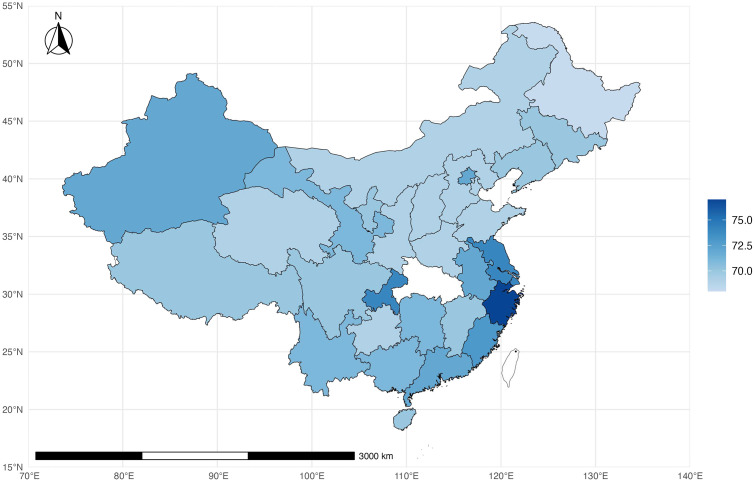
Distribution of average physical fitness scores across 30 provinces. Including body BMI, FVC (ml), Sit-and-reach(cm), 1-min sit-ups/pull-ups(number), standing long jump **(m)**, 50m sprint (sec), 50m × 8 round-trip running (sec), 800m/1000m(sec) and 1-minute rope skipping(number). Map rendered using public domain geographic data from Natural Earth (www.naturalearthdata.com). All map layers are CC0 licensed. Map generated with R (ggplot2 + sf).

S3 Table comprehensively summaries physical fitness metrics across different school levels and sexes. Overall, BMI and vital capacity show a clear upward trend as students’ progress through the educational stages from primary school to college. Boys generally perform better in physical tests such as the standing long jump, 50-meter sprint, and 1000-meter run, with their scores improving notably from middle school to college. On the other hand, girls demonstrate more robust performance in the sit-and-reach test across all school levels, with their flexibility improving with age. Regarding the one-minute sit-ups/pull-ups test, boys gradually increase their performance from middle school to college, whereas girls peak in middle school. Additionally, the total physical fitness scores reveal a decline for both boys and girls as they transition from primary school to higher education levels, indicating that overall fitness levels tend to decrease as students. The distribution of average physical fitness scores across 30 provinces is shown in Fig 3.

**Fig 4:**
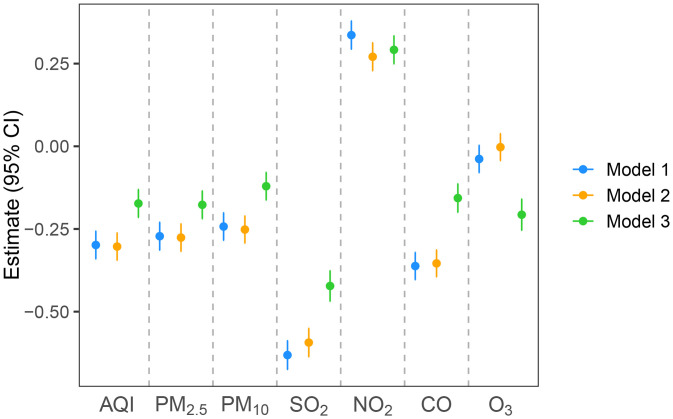
Association between AQI, air pollutants and physical fitness score in Chinese students aged 7-22 years across three models. model 1: adjusted for age (numeric variable), sex (categorical variable), residence (categorical variable), and province (categorical variable). model 2: further adjusted for moderate-to-vigorous physical activity and outdoor activity, muscle-strengthening exercise, parental support for physical activity, and parental preference for physical activity (all categorical variables). model 3: further adjusted for temperature (numeric variable) and humidity (numeric variable).

Fig 4 shows the result of the association between air pollution and physical fitness. In model 1, except for NO₂, all air pollutants and AQI were associated with the decreased physical fitness score. After adjusting for a comprehensive set of covariates, the negative associations persisted and remained statistically significant in model 2 and model 3. In model 3, we observed distinct associations between different categories of air pollutants and physical fitness. For AQI, it also shows an association with decreased physical fitness; every 1-unit increment in AQI concentration was associated with 0.17 points [CI: −0.21, −0.13]. Among the particulate matter pollutants, each 1 μg/m³ increase in PM_2.5_ and PM_10_ concentrations was associated with decreased physical fitness by 0.18 points [CI: −0.22, −0.14] and 0.12 [CI: −0.16, −0.08], respectively.

For gaseous pollutants, the analysis showed that SO_2_, O_3_, and CO also had a negative impact on physical fitness. Specifically, each 1 μg/m³ increase in SO_2_ was linked to 0.42 points [CI: −0.47, −0.38] decrease in physical fitness scores, while CO and O_3_ were associated with reductions of 0.16 points [CI: −0.20, −0.11] and 0.21 points [CI: −0.25, −0.16], respectively. In contrast, NO₂ was positively associated with physical fitness, with each 1-unit increase in NO₂ concentration linked to 0.29 points increase [CI: 0.25, 0.33], which is opposite to the effects observed for other pollutants.

In the sensitivity analyses, the results were consistent with the original findings, demonstrating the robustness of our conclusions. Using 3–5 years average AQI and air pollutants concentrations instead of 1-year averages yielded similar outcomes that all air pollutants and AQI were associated with the decreased physical fitness score. (S1–S3 Figs) suggesting that 1-year averages are reliable indicators of long-term air exposure. Subgroup analyses (S4–S6 Tables) indicated that these negative associations were generally stronger in boys than in girls, in rural than in urban students, and in middle/high school students compared with primary school children and college students.

## 4. Discussion

This nationally representative cross-sectional study reveals that increased ambient AQI levels and exposure to air pollutants are significantly associated with reduced physical fitness scores. These findings were consistent across all adjustment models, underscoring the detrimental impact of environmental pollution on physical fitness, with the exception of NO₂, which showed a positive association that persisted even after stratified analyses by age, sex, and region. Notably, the positive association with NO₂ was more evident in primary and junior high school students, and was stronger among girls than boys. Although some observed Estimate were modest, for instance, a 1 μg/m³ increase in PM_2.5_ reduced physical fitness scores by 0.18 point, but these small changes can be meaningful at the population level. In urban China, pollution exposure is widespread and chronic, so even slight reductions in fitness can add to a substantial public health burden when millions of children and adolescents are affected.

Particulate matter (PM_2.5_ and PM_10_) exhibited consistent negative associations with physical fitness across all models, with PM_2.5_ showing a more pronounced effect. PM₂.₅ can penetrate deep into alveoli, enter the bloodstream, and trigger systemic inflammation, impairing lung capacity (vital capacity test), slowing recovery in endurance events (800/1000 m run), and reducing muscular endurance (sit-ups, pull-ups) [27]. Prolonged exposure to PM_10_, although larger in size, contributes to respiratory conditions such as asthma and bronchitis, impairing exercise capacity and endurance. Elevated PM_10_ levels are associated with declining respiratory health [28], reduced physical performance, and even affected athletic performance [29]. Additionally, exposure to PM_10_ triggers systemic inflammation and oxidative stress [30], further compromising overall health. Both PM_2.5_ and PM_10_ have been linked to prevent people from engaging in regular physical activity and promote sedentary behavior [31–33]. Moreover, increased exposure to particulate matter has been linked to a higher risk of obesity [34], leading to elevated BMI levels, which compounding their negative impact on physical fitness. These findings align with previous studies highlighting the role of pulmonary and cardiovascular conditions in reducing physical activity levels [35,36] and overall health [37,38].

Most gaseous pollutants (SO₂, O₃, CO) demonstrated significant negative associations with physical fitness. SO₂ is strongly associated with bronchoconstriction, reduced oxygen transport, and cardiovascular strain, directly impairing endurance running, shuttle runs, and rope skipping [39,40] and playing a central role in fog-haze formation [41]. Prolonged exposure to O_3_ and PM_10_ has been shown to significantly increases diastolic blood pressure and reduces lung function [42]. O₃ exposure was linked to respiratory mortality, new-onset asthma, and exacerbation of existing respiratory conditions [43,44]. O₃ is a strong oxidant gas; experimental studies show that acute O₃ exposure during exercise increases airway resistance, decreases FEV₁/FVC, and elevates diastolic blood pressure. Such effects directly impair aerobic endurance and cyclic whole-body activities [45,46]. CO binds hemoglobin to form carboxyhemoglobin, reducing arterial oxygen content and shifting the oxyhemoglobin dissociation curve leftward, which decreases VO₂max and time-to-exhaustion in endurance events [47].These adverse health outcomes collectively impact performance in physical fitness tests such as the 800m/1000m run, 50m x 8 shuttles run, and 1-minute rope skipping, which require optimal cardiovascular and respiratory function [48,49].

In contrast, NO₂ exposure demonstrated an unexpected positive association with physical fitness scores, in contrast to the negative associations observed for most other air pollutants. This relationship persisted after multivariable adjustment, suggesting that the finding is unlikely to be explained solely by residual confounding from measured covariates. Subgroup analyses further revealed that this positive association was more pronounced among younger students, urban residents, and particularly girls. From a physiological perspective, girls generally have lower vital capacity and maximal voluntary ventilation compared with age-matched boys [50], leading to a higher proportion of pollutant deposition per unit of inspired air in the airways and alveoli under the same exposure level. From a behavioral perspective, boys typically engage in longer durations of outdoor physical activity than girls [51], potentially increasing their cumulative exposure dose and duration in high-NO₂ environments, thereby making harmful effects more likely to outweigh any indirect benefits from O₃ suppression. A further explanation involves the atmospheric chemistry of urban environments: in dense urban microenvironments, elevated NO₂ can titrate ambient O₃, reducing exposure to this potent respiratory oxidant during school hours [52]. This chemical interaction may partially attenuate O₃-related decrements in lung function, endurance, and aerobic performance, producing an apparent positive NO₂–physical fitness relationship in single-pollutant models. Additionally, age-related physiological plasticity may play a role; previous research has reported no significant association between NO₂ and lung function in youth, and adolescents may exhibit compensatory capacity under moderate exposure [53]. These explanations are consistent with the stronger NO₂ associations observed in urban settings and younger age groups, where background O₃ suppression and infrastructure differences are more pronounced. The presence of these air pollutants in the air not only reduces exercise capacity but also exacerbates pre-existing respiratory conditions. Collectively, the health complications arising from exposure to these pollutants inevitably lead to a decline in overall health, contributing to decreased physical fitness. This underscores the critical importance of stringent air quality control measures to protect public health, particularly for populations exposed to elevated levels of these harmful pollutants.

Based on our analysis of particulate matter (PM_2.5_, PM_10_) and gaseous pollutants (SO₂, O₃, CO), this study highlights the critical need to reduce AQI levels to mitigate the adverse health effects of air pollution. As AQI reflects the combined impact of particulate and gaseous pollutants, it serves as a robust indicator of environmental pollution’s overall effect on physical fitness. Elevated AQI levels are strongly linked to impaired lung function, systemic inflammation, and worsening cardiovascular and respiratory health. Reducing AQI is therefore essential, as both particulate and gaseous pollutants significantly contribute to these health risks. Implementing stricter air quality controls and targeted interventions is crucial to safeguard public health and improve physical fitness, particularly among vulnerable populations. These findings align with the WHO Global Air Quality Guidelines (2021) [54], which emphasize that no safe threshold exists for most air pollutants and that both particulate and gaseous pollutants contribute to cardiovascular and respiratory morbidity and mortality.

With its large sample size, this study enabled us to investigate the associations between environmental pollutants and physical fitness scores with enhanced statistical precision. The extensive geographic coverage and stratified random sampling method ensured a sample that accurately represents Chinese children, adolescents, and young adults. A key strength of this study is the comprehensive collection of data on various potential confounders. Notably, we adjusted our analysis for several factors, including moderate-to-vigorous physical activity, outdoor activities, muscle-strengthening exercises, parental support for exercise, and parental exercise preferences, to ensure the robustness of our results.

However, despite these strengths, some limitations should be acknowledged. First, the cross-sectional design limits causal inference, and longitudinal studies are needed to clarify temporal relationships. Second, exposure misclassification may exist, as satellite-based pollution data have been validated against ground-monitoring and provide reasonable population-level estimates but cannot fully capture individual-level exposure variability [55]. Moreover, survey-period exposures and testing dates may not be perfectly aligned at the monthly or seasonal scale; such non-differential temporal misalignment would generally bias estimates toward the null. Third, residual confounding from unmeasured factors such as built environment, road infrastructure, and socioeconomic indicators may remain. Fourth, geographic clustering and spatially structured pollution may leave residual spatial autocorrelation that understates standard errors; using region-clustered standard errors or spatial models would strengthen inference. Fifth, pollutants are correlated (all VIFs < 2), so collinearity appears limited but can still inflate variances and destabilize co-pollutant models; accordingly, single-pollutant estimates should be read as mixture/shared-source signals rather than pollutant-specific effects. Future research should improve exposure assessment by integrating satellite and ground data or by deploying personal exposure monitors in validation subsamples across seasons. Studies should adopt longitudinal designs with repeated and well-aligned exposure and outcome measurements. Broader contextual variables and school environment features should be included, for example neighborhood walkability, access to sports facilities, household and school socioeconomic conditions, traffic density, and green space. Methods that address correlated and spatially structured exposures are also needed, including mixture or dimension-reduction approaches, penalized regression, spatial models, and sensitivity analyses to alternative exposure windows and lags. Although the questionnaires used in this study have been validated and are widely used [56], some covariates were self-reported and may be affected by recall, misinterpretation, or social desirability; future waves should incorporate objective indicators or validation subsamples to quantify and correct potential measurement error. Finally, subgroup analyses should be expanded by age, sex, urbanicity, and geographic region to identify vulnerable populations and inform targeted public-health interventions.

## 5. Conclusion

This study comprehensively analyses the associations between AQI, air pollutants and physical fitness of Chinese students aged 7–22 years. Except for NO₂, most environmental pollutants showed significant negative associations with physical fitness, even after controlling for exercise conditions, temperature, and humidity. Interestingly, NO₂ showed a positive association, particularly stronger in girls than in boys, and more evident among younger students and urban residents, which may reflect non-causal mechanisms related to atmospheric chemistry, physiological differences, and activity patterns. These findings translate into actionable pathways for public health and sustainable development, including integrating routine school fitness testing with local air-quality information to enable early detection and non-stigmatizing feedback, adapting educational practice by adjusting outdoor physical education on high-pollution days, scheduling activities at cleaner times and providing well-ventilated indoor alternatives, and strengthening policy and infrastructure around schools by managing nearby emissions, improving ventilation and filtration, and sharing real-time environmental information. Furthermore, physical fitness assessments can serve as early indicators of environmental health risks in youth. To embed this within school health monitoring, conduct semesterly assessments, interpret results alongside local air-quality data, apply simple age- and sex-specific thresholds to trigger school actions, and ensure privacy-safe follow-up and parent communication. Future research should use longitudinal designs with repeated, well-aligned measures and enhanced exposure assessment to test interventions and quantify benefits. Future research should focus on longitudinal studies to establish causality and explore interventions to mitigate the impact of air pollution on physical fitness.

## Supporting information

S1 Table General characteristics of study participants. Continuous variables are shown as means (SD), and categorical variables are shown as numbers (percentages).
(DOCX)

S2 Table Distributions of AQI, air pollutants, temperature, and humility. AQI, Air Quality Index; PM2.5, particulate matter with an aerodynamic diameter of ≤2.5 μm; PM10, particulate matter with an aerodynamic diameter of ≤1 μm; PM10, particulate matter with an aerodynamic diameter of ≤10 μm; SO2, sulfur dioxide; NO2, nitrogen dioxide; CO, carbon monoxide; O_3_, ozone.
(DOCX)

S3 Table Summary of physical fitness across different school levels and sex. Missing values in specific columns (indicated by “-”) represent tests that were not conducted for the respective group.
(DOCX)

S4 Table Subgroup analysis by grade. (DOCX)

S5 Table Subgroup analysis by region. (DOCX)

S6 Table Subgroup analysis by sex. (DOCX)

S1 Fig Association between 3-years average AQI, air pollutants and physical fitness score in Chinese students aged 7–22 years across three models. Association between 3-years average AQI, air pollutants and physical fitness score in Chinese students aged 7–22 years across three models.
(PDF)

S2 Fig Association between 4-years average AQI, air pollutants and physical fitness score in Chinese students aged 7–22 years across three models. Association between 4-years average AQI, air pollutants and physical fitness score in Chinese students aged 7–22 years across three models.
(PDF)

S3 Fig Association between 5-years average AQI, air pollutants and physical fitness score in Chinese students aged 7–22 years across three models. Association between 5-years average AQI, air pollutants and physical fitness score in Chinese students aged 7–22 years across three models.
(PDF)

S1 Text Protocols for Physical Fitness Tests. (DOCX)
