# Supplementary material for: Is air pollution negatively associated with physical fitness?—A cross-sectional study in 174,246 Chinese students
Source: PLoS One. 2025 Nov 6;20(11):e0336417. doi: 10.1371/journal.pone.0336417 (PMC12591427; doi:10.1371/journal.pone.0336417)
Supplement: S1 Table — Continuous variables are shown as means (SD), and categorical variables are shown as numbers (percentages). (DOCX) [file pone.0336417.s001.docx]

| **Table S1** General characteristics of study participants (N=174,036). | | |
| --- | --- | --- |
| Variables | Group | Sample Size |
| **Demographic Information** | | |
| Sex | Boys | 99,407 (57.1%) |
|  | Girls | 74,629 (42.9%) |
| Residence | Urban | 83,384(47.9%) |
|  | Rural | 90,652(52.1%) |
| Height (cm) |  | 158.65 ± 13.32 |
| Weight (kg) |  | 49.75 ± 13.63 |
| Age (years) |  | 15.13 ± 3.93 |
| **Exercise** **Situation** |  |  |
| Moderate-To-Vigorous Physical Activity | ≥ 1 hour per day | 142,409 (81.8%) |
|  | < 1 hour per day | 31,627 (18.2%) |
| Outdoor Activities | ≥ 2 hours per day | 163,323 (93.8%) |
|  | < 2 hours per day | 10,713 (7.2%) |
| Muscle-Strengthening Exercises | ≥ 2 days per week | 53,421 (30.7%) |
|  | < 2 days per week | 120,615 (69.3%) |
| Parental Support for Exercise | Supportive | 140,238 (80.6%) |
|  | Neutral | 28,407 (16.3%) |
|  | Unsupportive | 5,391 (3.1%) |
| Parental Preference for Exercise | Both like | 80,229(46.1%) |
|  | Father like | 32,934(18.9%) |
|  | Mother like | 17,164(9.9%) |
|  | Both don't like | 43,709(25.1%) |
| **Note:** Continuous variables are shown as means (SD), and categorical variables are shown as numbers (percentages). All data presented in this table are aggregated at the group level. No individual-level information is included. All variables are fully anonymized and cannot be used to identify individual participants. The dataset complies with the journal's data sharing policy and participant privacy requirements. | | |
