# Supplementary material for: Is air pollution negatively associated with physical fitness?—A cross-sectional study in 174,246 Chinese students
Source: PLoS One. 2025 Nov 6;20(11):e0336417. doi: 10.1371/journal.pone.0336417 (PMC12591427; doi:10.1371/journal.pone.0336417)
Supplement: S2 Table — AQI, Air Quality Index; PM2.5, particulate matter with an aerodynamic diameter of ≤2.5 μm; PM10, particulate matter with an aerodynamic diameter of ≤1 μm; PM10, particulate matter with an aerodynamic diameter of ≤10 μm; SO2, sulfur dioxide; NO2, nitrogen dioxide; CO, carbon monoxide; O3, ozone. (DOCX) [file pone.0336417.s002.docx]

| **Table S2** Distributions of AQI, air pollutants, temperature, and humility | | | | | | | | | |
| --- | --- | --- | --- | --- | --- | --- | --- | --- | --- |
| **Province** | **AQI** | **PM_2.5_** | **PM_10_** | **SO_2_** | **NO_2_** | **CO** | **O_3_** | **Temperature** | **Humidity** |
| **Anhui** | 50.62 | 76.34 | 7.27 | 43.99 | 0.82 | 48.83 | 0.15 | 0.78 | 74.75 |
| **Beijing** | 18 | 23.92 | 5.97 | 10.03 | 0.54 | 39.78 | 0.08 | 0.55 | 73.25 |
| **Chongqing** | 41.23 | 62.11 | 7.16 | 41.33 | 0.9 | 24.25 | 0.16 | 0.8 | 56.00 |
| **Fujian** | 25.44 | 42.88 | 6.92 | 21.87 | 0.64 | 64.74 | 0.21 | 0.65 | 54.50 |
| **Gansu** | 38.72 | 74.9 | 16.37 | 43.09 | 1.01 | 39.7 | 0.08 | 0.69 | 59.50 |
| **Guangdong** | 34.59 | 60.08 | 8.36 | 36.96 | 0.8 | 73.23 | 0.28 | 0.85 | 87.25 |
| **Guangxi** | 38.55 | 64.28 | 12.92 | 29.89 | 0.95 | 53.35 | 0.2 | 0.69 | 60.50 |
| **Guizhou** | 33.22 | 46.76 | 6.37 | 17.32 | 0.72 | 45.21 | 0.15 | 0.8 | 56.25 |
| **Hainan** | 20.52 | 37.16 | 4.49 | 11.12 | 0.67 | 88.43 | 0.24 | 0.77 | 77.75 |
| **Hebei** | 73.77 | 128.73 | 15.51 | 56.04 | 1.34 | 36 | 0.11 | 0.61 | 60.89 |
| **Heilongjiang** | 48.83 | 69.17 | 18.75 | 31.49 | 0.77 | 40.17 | 0 | 0.64 | 56.00 |
| **Henan** | 69.54 | 104.84 | 9.99 | 45.94 | 1.05 | 49.01 | 0.13 | 0.6 | 84.67 |
| **Hunan** | 49.74 | 70.29 | 9.96 | 33.64 | 0.95 | 55.99 | 0.16 | 0.71 | 68.75 |
| **Inner Mongolia** | 60.77 | 99.88 | 19.19 | 43.93 | 1.29 | 40.27 | 0.04 | 0.56 | 58.50 |
| **Jiangsu** | 51.22 | 86.5 | 8.28 | 44.51 | 0.89 | 48.76 | 0.15 | 0.72 | 72.50 |
| **Jiangxi** | 46.03 | 76.9 | 12.95 | 35.25 | 0.99 | 54.97 | 0.18 | 0.64 | 63.50 |
| **Jilin** | 46.54 | 69.44 | 15.72 | 33.33 | 0.96 | 39.92 | 0.04 | 0.6 | 61.25 |
| **Liaoning** | 52.78 | 83.88 | 23.25 | 39.36 | 1.02 | 41.07 | 0.06 | 0.65 | 60.25 |
| **Ningxia** | 37.56 | 69.97 | 14.07 | 35.9 | 0.87 | 47.93 | 0.05 | 0.56 | 71.75 |
| **Qinghai** | 29.53 | 46.73 | 14.65 | 31.73 | 1.1 | 55.93 | 0.03 | 0.61 | 77.25 |
| **Shaanxi** | 55.93 | 83.12 | 9.12 | 38.91 | 1.02 | 27.85 | 0.11 | 0.7 | 71.00 |
| **Shandong** | 58.28 | 100.08 | 13.68 | 39.56 | 0.86 | 55.75 | 0.12 | 0.61 | 69.50 |
| **Shanghai** | 36.31 | 49.06 | 6.97 | 45.38 | 0.7 | 57.48 | 0.17 | 0.75 | 64.50 |
| **Shanxi** | 63.29 | 111.22 | 22.17 | 45.97 | 1.33 | 41.81 | 0.09 | 0.56 | 39.25 |
| **Sichuan** | 46.7 | 68.45 | 7.09 | 38.54 | 0.81 | 29.16 | 0.14 | 0.87 | 80.50 |
| **Tianjin** | 60.92 | 85.19 | 10.81 | 52.18 | 1.09 | 41.42 | 0.11 | 0.6 | 64.50 |
| **Tibet** | 21.11 | 39.68 | 4.7 | 14.39 | 1.01 | 43.72 | 0.07 | 0.39 | 80.00 |
| **Xinjiang** | 75.15 | 129.14 | 9.46 | 43.75 | 1.31 | 40.08 | 0.06 | 0.58 | 70.25 |
| **Yunnan** | 19.03 | 33.27 | 6.55 | 17.34 | 0.72 | 46.96 | 0 | 0.73 | 61.00 |
| **Zhejiang** | 36.94 | 66.01 | 7.3 | 43.88 | 0.79 | 48.1 | 0.16 | 0.72 | 64.00 |

Notes: AQI, Air Quality Index; PM2.5, particulate matter with an aerodynamic diameter of ≤2.5 μm; PM10, particulate matter with an aerodynamic diameter of ≤1 μm; PM10, particulate matter with an aerodynamic diameter of ≤10 μm; SO2, sulfur dioxide; NO2, nitrogen dioxide; CO, carbon monoxide; O_3_, ozone. All data presented are aggregated at the provincial level and do not include any personally identifiable information.
