# Supplementary material for: Is air pollution negatively associated with physical fitness?—A cross-sectional study in 174,246 Chinese students
Source: PLoS One. 2025 Nov 6;20(11):e0336417. doi: 10.1371/journal.pone.0336417 (PMC12591427; doi:10.1371/journal.pone.0336417)
Supplement: S3 Table — Missing values in specific columns (indicated by “-”) represent tests that were not conducted for the respective group. (DOCX) [file pone.0336417.s003.docx]

| **Table S3** Summary of physical fitness across different school levels and sex | | | | | | | | |
| --- | --- | --- | --- | --- | --- | --- | --- | --- |
|  |  | Boys |  |  |  | Girls |  |  |
|  | Primary School | Middle School | High School | College | Primary School | Middle School | High School | College |
| BMI | 16.75 ± 1.93 | 18.60 ± 2.23 | 20.84 ± 2.86 | 21.67 ± 2.95 | 16.59 ± 2.02 | 19.12 ± 2.45 | 21.00 ± 2.72 | 20.69 ± 2.67 |
| FVC (ml) | 1806.35 ± 450.78 | 2833.80 ± 723.09 | 3731.73 ± 760.12 | 4009.62 ± 721.31 | 1665.16 ± 435.77 | 2192.68 ± 490.78 | 2420.52 ± 493.58 | 2682.83 ± 491.14 |
| Sit-and-reach (cm) | 5.57 ± 5.17 | 7.36 ± 6.11 | 11.08 ± 6.29 | 11.67 ± 6.23 | 9.73 ± 5.40 | 11.44 ± 5.81 | 14.10 ± 5.76 | 15.31 ± 5.81 |
| 1-min sit-ups/Pull-ups (number) | 32.09 ± 10.41 | 3.04 ± 4.22 | 4.66 ± 4.41 | 5.33 ± 4.72 | 28.36 ± 9.80 | 30.53 ± 9.30 | 32.11 ± 9.91 | 31.29 ± 8.97 |
| Standing Long Jump (cm) | - | 192.49 ± 26.85 | 220.56 ± 23.78 | 222.48 ± 21.45 | - | 153.84 ± 20.77 | 160.82 ± 19.40 | 163.74 ± 17.60 |
| 50m sprint (sec) | 9.49 ± 0.86 | 8.21 ± 0.78 | 7.61 ± 0.64 | 7.66 ± 0.63 | 10.04 ± 0.89 | 9.48 ± 0.83 | 9.51 ± 0.84 | 9.65 ± 0.83 |
| 50m×8 round-trip running (sec) | 116.99 ± 12.28 | - | - | - | 123.15 ± 12.75 | - | - | - |
| 800m/1000m Running (sec) | - | 272.43 ± 38.29 | 255.78 ± 29.32 | 263.98 ± 29.49 | - | 252.18 ± 31.80 | 251.94 ± 27.24 | 259.30 ± 27.73 |
| 1-minute Rope Skipping (number) | 94.78 ± 40.58 | - | - | - | 102.11 ± 35.35 | - | - | - |
| Total Score | 74.72 ± 7.96 | 71.64 ± 9.25 | 70.48 ± 8.71 | 66.06 ± 8.25 | 74.60 ± 7.80 | 72.03 ± 8.53 | 67.52 ± 9.29 | 68.04 ± 9.52 |
| **Note**: Missing values in specific columns (indicated by "-") represent tests that were not conducted for the respective group. This table only presents group-level summary statistics (means and standard deviations) stratified by sex and school level. No individual-level data or personally identifiable information is included. | | | | | | | | |
