# Supplementary material for: Is air pollution negatively associated with physical fitness?—A cross-sectional study in 174,246 Chinese students
Source: PLoS One. 2025 Nov 6;20(11):e0336417. doi: 10.1371/journal.pone.0336417 (PMC12591427; doi:10.1371/journal.pone.0336417)
Supplement: S4 Table — (DOCX) [file pone.0336417.s004.docx]

**Table S4** Subgroup analysis by grade

| Grade | Pollutant | Estimate | 95% CI Lower | 95% CI Upper | *P* |
| --- | --- | --- | --- | --- | --- |
| Primary school | AQI | -0.11 | -0.18 | -0.04 | 0.001 |
|  | PM2.5 | -0.08 | -0.15 | -0.01 | 0.026 |
|  | PM10 | -0.1 | -0.17 | -0.04 | 0.003 |
|  | SO2 | -0.69 | -0.77 | -0.61 | <0.001 |
|  | NO2 | 0.59 | 0.52 | 0.67 | <0.001 |
|  | CO | -0.31 | -0.38 | -0.24 | <0.001 |
|  | O3 | -0.4 | -0.48 | -0.33 | <0.001 |
| Middle school | AQI | -0.33 | -0.42 | -0.24 | <0.001 |
|  | PM2.5 | -0.35 | -0.43 | -0.26 | <0.001 |
|  | PM10 | -0.32 | -0.41 | -0.24 | <0.001 |
|  | SO2 | -0.48 | -0.58 | -0.38 | <0.001 |
|  | NO2 | 0.23 | 0.14 | 0.32 | <0.001 |
|  | CO | -0.26 | -0.35 | -0.18 | <0.001 |
|  | O3 | -0.1 | -0.2 | -0.01 | 0.028 |
| High school | AQI | -0.09 | -0.17 | -0.0 | 0.043 |
|  | PM2.5 | -0.15 | -0.24 | -0.07 | <0.001 |
|  | PM10 | 0.1 | 0.02 | 0.19 | 0.013 |
|  | SO2 | -0.3 | -0.4 | -0.21 | <0.001 |
|  | NO2 | 0.17 | 0.08 | 0.25 | <0.001 |
|  | CO | 0.08 | -0.01 | 0.17 | 0.065 |
|  | O3 | -0.24 | -0.34 | -0.15 | <0.001 |
| College | AQI | -0.27 | -0.36 | -0.17 | <0.001 |
|  | PM2.5 | -0.23 | -0.32 | -0.13 | <0.001 |
|  | PM10 | -0.32 | -0.42 | -0.21 | <0.001 |
|  | SO2 | -0.44 | -0.54 | -0.34 | <0.001 |
|  | NO2 | 0.06 | -0.03 | 0.15 | 0.176 |
|  | CO | -0.19 | -0.29 | -0.08 | <0.001 |
|  | O3 | -0.02 | -0.14 | 0.1 | 0.747 |
