# Supplementary material for: Is air pollution negatively associated with physical fitness?—A cross-sectional study in 174,246 Chinese students
Source: PLoS One. 2025 Nov 6;20(11):e0336417. doi: 10.1371/journal.pone.0336417 (PMC12591427; doi:10.1371/journal.pone.0336417)
Supplement: S5 Table — (DOCX) [file pone.0336417.s005.docx]

**Table S5** Subgroup analysis by region

| Region | Pollutant | Estimate | 95% CI Lower | 95% CI Upper | *P* |
| --- | --- | --- | --- | --- | --- |
| Rural | AQI | -0.22 | -0.28 | -0.16 | <0.001 |
|  | PM2.5 | -0.24 | -0.3 | -0.18 | <0.001 |
|  | PM10 | -0.13 | -0.19 | -0.07 | <0.001 |
|  | SO2 | -0.47 | -0.53 | -0.4 | <0.001 |
|  | NO2 | 0.2 | 0.14 | 0.26 | <0.001 |
|  | CO | -0.09 | -0.15 | -0.03 | 0.002 |
|  | O3 | -0.04 | -0.11 | 0.02 | 0.189 |
| Urban | AQI | -0.17 | -0.22 | -0.11 | <0.001 |
|  | PM2.5 | -0.15 | -0.21 | -0.09 | <0.001 |
|  | PM10 | -0.14 | -0.2 | -0.08 | <0.001 |
|  | SO2 | -0.41 | -0.48 | -0.34 | <0.001 |
|  | NO2 | 0.37 | 0.3 | 0.43 | <0.001 |
|  | CO | -0.26 | -0.32 | -0.2 | <0.001 |
|  | O3 | -0.38 | -0.45 | -0.31 | <0.001 |
