# Supplementary material for: Is air pollution negatively associated with physical fitness?—A cross-sectional study in 174,246 Chinese students
Source: PLoS One. 2025 Nov 6;20(11):e0336417. doi: 10.1371/journal.pone.0336417 (PMC12591427; doi:10.1371/journal.pone.0336417)
Supplement: S6 Table — (DOCX) [file pone.0336417.s006.docx]

**Table S6** Subgroup analysis by sex

| Sex | Pollutant | Estimate | 95% CI Lower | 95% CI Upper | *P* |
| --- | --- | --- | --- | --- | --- |
| Girls | AQI | -0.16 | -0.22 | -0.09 | <0.001 |
|  | PM2.5 | -0.11 | -0.18 | -0.05 | <0.001 |
|  | PM10 | -0.14 | -0.21 | -0.08 | <0.001 |
|  | SO2 | -0.5 | -0.57 | -0.43 | <0.001 |
|  | NO2 | 0.56 | 0.5 | 0.63 | <0.001 |
|  | CO | -0.2 | -0.27 | -0.13 | <0.001 |
|  | O3 | -0.52 | -0.6 | -0.45 | <0.001 |
| Boys | AQI | -0.19 | -0.24 | -0.14 | <0.001 |
|  | PM2.5 | -0.23 | -0.28 | -0.18 | <0.001 |
|  | PM10 | -0.11 | -0.17 | -0.06 | <0.001 |
|  | SO2 | -0.36 | -0.42 | -0.3 | <0.001 |
|  | NO2 | 0.08 | 0.03 | 0.14 | 0.004 |
|  | CO | -0.13 | -0.19 | -0.07 | <0.001 |
|  | O3 | 0.04 | -0.02 | 0.1 | 0.209 |
