# Supplementary figures and images for: Is air pollution negatively associated with physical fitness?—A cross-sectional study in 174,246 Chinese students
Source: PLoS One. 2025 Nov 6;20(11):e0336417. doi: 10.1371/journal.pone.0336417 (PMC12591427; doi:10.1371/journal.pone.0336417)

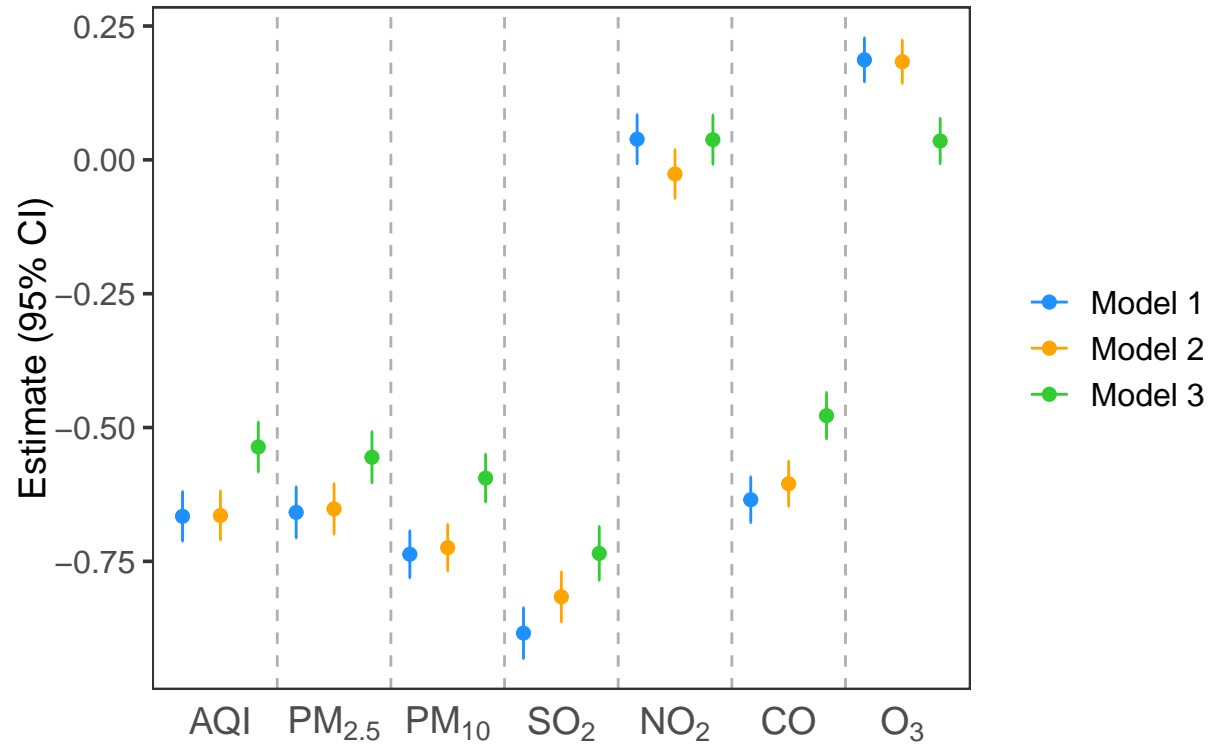

Supplement: S1 Fig — Association between 3-years average AQI, air pollutants and physical fitness score in Chinese students aged 7–22 years across three models. (PDF) [file pone.0336417.s007.pdf]

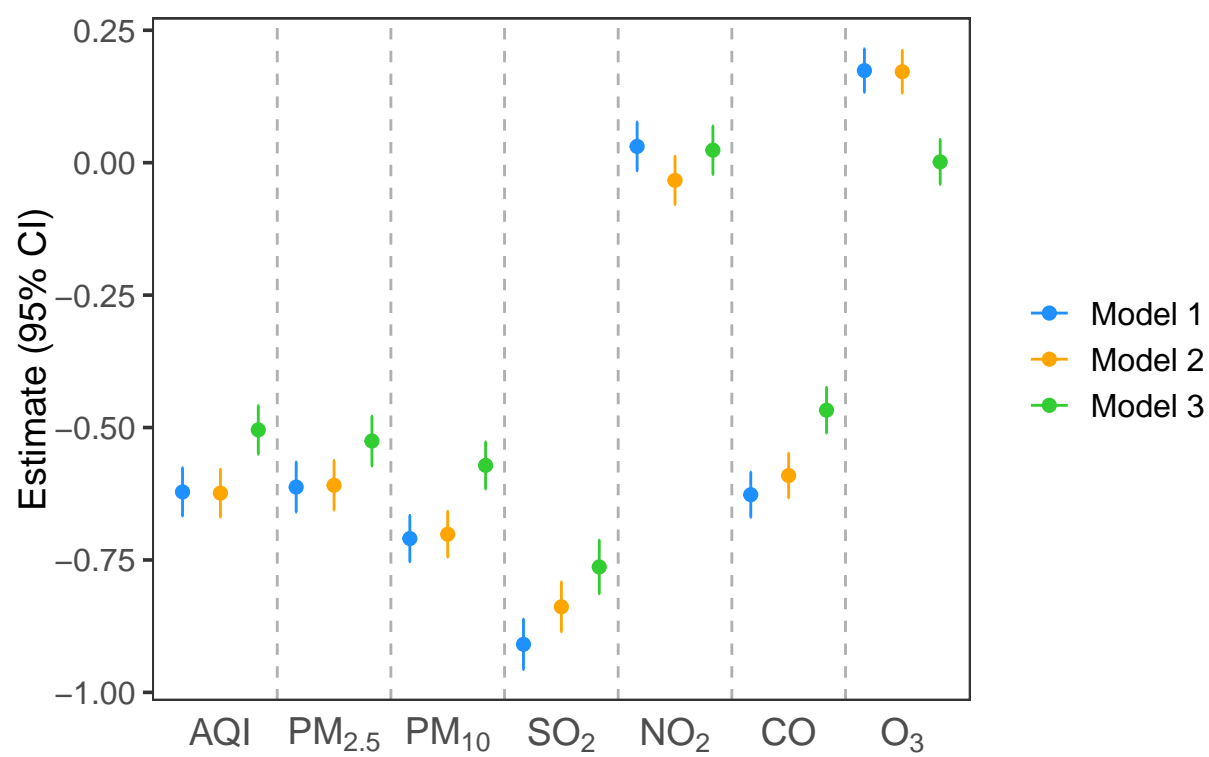

Supplement: S2 Fig — Association between 4-years average AQI, air pollutants and physical fitness score in Chinese students aged 7–22 years across three models. (PDF) [file pone.0336417.s008.pdf]

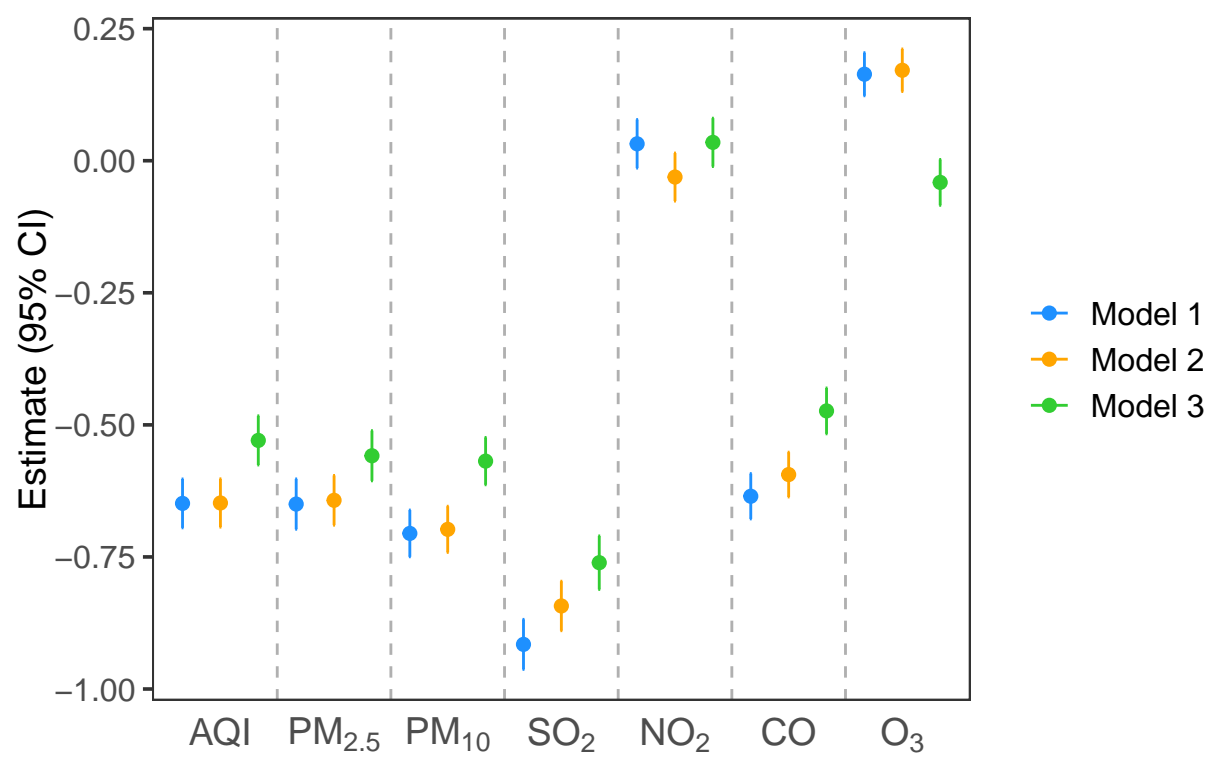

Supplement: S3 Fig — Association between 5-years average AQI, air pollutants and physical fitness score in Chinese students aged 7–22 years across three models. (PDF) [file pone.0336417.s009.pdf]
