# Supplementary material for: Is air pollution negatively associated with physical fitness?—A cross-sectional study in 174,246 Chinese students
Source: PLoS One. 2025 Nov 6;20(11):e0336417. doi: 10.1371/journal.pone.0336417 (PMC12591427; doi:10.1371/journal.pone.0336417)
Supplement: S1 Text — (DOCX) [file pone.0336417.s010.docx]

Text S1 Protocols for Physical Fitness Tests.

Text S1 provides detailed descriptions of the standardized protocols used for each physical fitness test conducted in this study:

(1) BMI test

The participant stands barefoot on the platform, and weight is recorded after the prompt. Heels, sacrum, and scapula must stay in contact with the measurement column, maintaining an upright posture with the head straight and avoiding viewing the data. Height and weight are measured in a single attempt. BMI is calculated as weight (kg) / height (m²) ², and school-based BMI assessments are a potential tool for addressing adolescent obesity[1].

(2) FVC test

The forced vital capacity test is conducted using an electronic spirometer placed on a stable surface in a well-ventilated room with a power supply. The tester switches on the device and waits for the display to stabilize at "0," indicating readiness. A mouthpiece is attached to the spirometer tube and handed to the participant, along with instructions to stay relaxed during the test. The participant stands naturally, holding the tube handle with the pressure tube positioned above the spirometer. With their head slightly tilted back, they inhale deeply to full lung capacity and then exhale slowly and completely through the mouthpiece. The highest value displayed (in milliliters, without decimals) is recorded after two trials, with a maximum interval of 15 seconds between tests. The purpose of this test is to evaluate lung function[2].

(3) Sit-and-reach test

The sit-and-reach test is conducted using a sit-and-reach box and a mat placed on a flat surface. The participant sits on the mat facing the box with legs extended straight, heels together against the footplate, and toes naturally spaced about 10–15 cm apart. During the test, the participant keeps their knees straight, extends both hands forward with palms down, and uses the middle fingers to push the slider forward smoothly until they reach their limit. If the slider moves past the "0" mark, the score is recorded as positive; if not, it is recorded as negative. Each participant performs the test twice, with the best score recorded in centimeters to one decimal place. This test is purpose on evaluates flexibility[3].

(4) 1-min sit-ups (primary school students in grades 3 to 6 and girls above primary school) / pull-ups test (boys above primary school)

Pull-ups: The pull-up test is conducted using a high bar or horizontal bar, with the bar's thickness chosen to ensure a firm grip. The participant stands naturally facing the bar, then jumps to grip it with an overhand grip, hands shoulder-width apart, and hangs with arms fully extended. Once the body is steady, the participant pulls up until the chin is above the bar, then returns to the initial hanging position with arms fully extended. This completes one pull-up. No additional body movements are allowed during the pull-up.

Sit-ups: The participant lies on a mat with knees bent at 90 degrees and arms crossed behind the head. They perform as many as possible in a minute, ensuring the elbows reach past the knees and the shoulders touch the mat between each repetition. This test is to assess the endurance and strength of the core muscles[4].

(5) Standing long jump test (students above primary school)

The participant should stand naturally, feet apart, behind the starting line without stepping over it. From a stationary position, jump as far as possible, ensuring no extra steps or hops are taken. Perform three attempts and record the longest jump. This test can be a useful tool to assess lower-body muscular power[5,6].

(6) 50m sprint

This test requires a minimum of two participants, who start from a designated line. At the signal "Go" accompanied by a flag wave, the participants initiate their run. The timer starts as the flag is waved and stops once the participant’s torso crosses the plane of the finish line. This test is widely used to assess an individual's acceleration and speed capabilities[7].

(7) 50m×8 round-trip running (primary school students in grade 5 to 6)

Participants begin at the starting line. At the signal, they run 50 meters to the opposite end, touch the line, and turn back. They repeat this process for a total of eight round trips, covering 400 meters. Timing stops once they complete the final lap and cross the starting line. This test was to analyze the temporal trends in cardiorespiratory fitness[8].

(8) 1000m run (boys above primary school) / 800m run (girls above primary school)

After identity verification and receiving numbered running vests, participants are grouped into sets of 20 to 30. They are directed to the starting point, where the judge provides instructions and positions them at the start line. At the command "On your marks, get set, go," the timer starts, and participants begin the run. The judge records the final time once the participant crosses the finish line. This test is to assess the temporal trends in cardiorespiratory fitness[8].

(9) 1-minute rope skipping (primary school students)

The participant adjusts the length of the rope to a suitable height. Upon hearing the start signal, they begin skipping with the standard technique: swinging the rope forward and jumping with both feet together. Each jump, combined with a full rotation of the rope, counts as one repetition. When the stop signal is given, the participant ceases skipping. The tester counts and records the total number of skips completed within one minute. This test is to assess an individual's coordination, cardiorespiratory endurance, and lower limb explosive power[9].

References

1. Nihiser AJ, Lee SM, Wechsler H, McKenna M, Odom E, Reinold C, et al. BMI measurement in schools. Pediatrics. 2009;124 Suppl 1: S89-97. doi:10.1542/peds.2008-3586L

2. Tojo N. [pulmonary function testing: History up to the present]. Rinsho Byori. 2014;62: 1205–1211.

3. Wilder RP, Greene JA, Winters KL, Long WB, Gubler K, Edlich RF. Physical fitness assessment: An update. J Long Term Eff Med Implants. 2006;16: 193–204. doi:10.1615/jlongtermeffmedimplants.v16.i2.90

4. Bianco A, Lupo C, Alesi M, Spina S, Raccuglia M, Thomas E, et al. The sit up test to exhaustion as a test for muscular endurance evaluation. Springerplus. 2015;4: 309. doi:10.1186/s40064-015-1023-6

5. Marin-Jimenez N, Perez-Bey A, Cruz-Leon C, Conde-Caveda J, Segura-Jimenez V, Castro-Piñero J, et al. Criterion-related validity and reliability of the standing long jump test in adults: The adult-fit project. Eur J Sport Sci. 2024;24: 1379–1392. doi:10.1002/ejsc.12182

6. Fernandez-Santos JR, Ruiz JR, Cohen DD, Gonzalez-Montesinos JL, Castro-Piñero J. Reliability and validity of tests to assess lower-body muscular power in children. J Strength Cond Res. 2015;29: 2277–2285. doi:10.1519/JSC.0000000000000864

7. International physical fitness test. In: The Sport Journal [Internet]. 11 Feb 2008 [cited 26 Dec 2024]. Available: https://thesportjournal.org/article/international-physical-fitness-test/

8. Li C, Zhang J, Maisiwute M, Zhou Z, Zang L. Temporal trends and distributional changes in cardiorespiratory fitness among Chinese children and adolescents from 1985 to 2019. Scand J Med Sci Sports. 2024;34: e14669. doi:10.1111/sms.14669

9. Trecroci A, Cavaggioni L, Caccia R, Alberti G. Jump rope training: Balance and motor coordination in preadolescent soccer players. J Sports Sci Med. 2015;14: 792–798.
